# Strategies for overcoming data scarcity, imbalance, and feature selection challenges in machine learning models for predictive maintenance

**DOI:** 10.1038/s41598-024-59958-9

**Published:** 2024-04-26

**Authors:** Ali Hakami

**Affiliations:** Mechanical and Industrial Engineering Department, College of Engineering and Computing in Al-Gunfudha, Umm Al-Qura University, 21961 Mecca, Saudi Arabia

**Keywords:** Engineering, Mechanical engineering

## Abstract

Predictive maintenance harnesses statistical analysis to preemptively identify equipment and system faults, facilitating cost- effective preventive measures. Machine learning algorithms enable comprehensive analysis of historical data, revealing emerging patterns and accurate predictions of impending system failures. Common hurdles in applying ML algorithms to PdM include data scarcity, data imbalance due to few failure instances, and the temporal dependence nature of PdM data. This study proposes an ML-based approach that adapts to these hurdles through the generation of synthetic data, temporal feature extraction, and the creation of failure horizons. The approach employs Generative Adversarial Networks to generate synthetic data and LSTM layers to extract temporal features. ML algorithms trained on the generated data achieved high accuracies: ANN (88.98%), Random Forest (74.15%), Decision Tree (73.82%), KNN (74.02%), and XGBoost (73.93%).

## Introduction

The advent of Industry 4.0, as defined by^1,2^, has revolutionized traditional manufacturing processes and business models. Industry 4.0 represents the fourth industrial revolution, integrating digital technologies, automation, data exchange, and the Internet of Things (IoT) to transform traditional manufacturing processes into interconnected and intelligent production systems. In the era of Industry 4.0, unplanned downtime’s are of major concern because they are believed to cause high drops in productivity. According to^3^ unplanned downtime roughly accounts for 5–20% of such drops. A proactive maintenance approach is therefore crucial for minimizing unplanned downtime, making the most out of available resources, and increasing output^1^. Predictive maintenance(PdM) has proven to be such an approach. It entails the use of statistical analysis of historical data to identify eminent faults in equipment and systems before time. This way, maintenance operations can be carried out in time hence reducing unexpected stops.

The use of machine learning algorithms in data analytics has seen rapid growth over the recent past^4^. In PdM, such algorithms enable the analysis of past occasions, the identification of emerging patterns, and the accurate prediction of future outcomes based on the acquired insight^5–7^. Such insights simplify maintenance procedures and consequently improve resource utilization^8^. Moreover, predictive maintenance datasets involve many crucial variables like device importance, accessible resources, and operational limitations^9^. This makes ML techniques more desirable as they are fast and can seamlessly process vast parameters delivering insights in time, enabling timely preparation for potential breakdowns^10^. Besides conventional machine learning methods, the application of deep learning (DL) and reinforcement learning (RL) has become a major area of research in PdM^11^. With reinforcement learning machines perfect maintenance processes, and can perform them autonomously resulting in improved performance and efficiency^12^.

Two common outcomes of interest in PdM are fault diagnosis and categorization^13,14^, and Remaining Useful Life (RUL) prediction. In fault diagnosis, algorithms learn the normal patterns from the training data and then flag any observations that significantly deviate from the learned patterns^15^. Such observations are thought of as indicative of abnormalities in the system and possibly impending failure. For RUL, the algorithms learn to predict the remaining useful life of a component from its performance. With these two maintenance decisions can be made in time, either manually or by a decision-making agent trained through reinforcement.

As academics and practitioners strive to optimize maintenance methods and avoid costly equipment failures by using data and artificial intelligence, various research literature on the application of ML in PdM has emerged^16,17^. Due to the diversity of machinery in industries and their performance patterns during different faulty conditions, PdM datasets tend to be diverse. Specializations therefore play a significant role in model choice. For instance, Random Forest (RF) algorithms work better with independent observations, while Long Short-Term Memory (LSTM) neural networks have proven effective with sequential data such as time series^18–20^. The numerous literatures test different ML models in numerous business settings to establish their adaptability and efficacy^9,18^. Researchers have identified common problems while dealing with different case studies. Data scarcity, feature selection from abundant sensor data properties, data imbalance, and temporal relationships in data are among these difficulties^21–23^.

Machine learning approaches, especially deep learning, typically require large datasets to adequately learn failure patterns. The availability of such data can be a challenge, especially for new systems or components where there is insufficient historical data. Several reviewed literature have reported this challenge. For instance, Ganesan et al.^24^, who employed specialized modeling where different models were developed with data from each component of interest, acknowledge the challenge as a limitation to the approach. Rojek et al.^25^ encountered challenges related to data availability, reliability, and completeness while applying AI techniques in PdM. Another study^26^. Attempted to address data scarcity by employing simulation to generate PdM data for automotive engine components. However, they further encountered issues with computational cost and feasibility in real-world applications. Data imbalance is another challenge that arises from the fact that there are typically few failure instances in maintenance datasets. Models trained on such datasets often do not have enough failure cases to learn from. Susto et al.^16^. Addressed this issue by introducing prediction horizons, where the most recent ’m’ instances in each run are segregated as failures and the preceding ’n-m’ instances as non-failures. The temporal nature of sensor data, where readings are collected over time, also presents complexities due to sequential dependence between readings. This can impact common feature extraction types such as those involving statistical moments. Another study^13,27^ proposes the use of specialized networks such as GRUs and LSTMs during feature extraction to learn the temporal patterns. Such feature learnings also help avoid feature selection which can degrade data quality. A research gap exists concerning the need for a robust PdM approach that addresses the challenges of data scarcity, temporal dependence in sensor data, data imbalance, and limitations of feature selections. This study proposes a machine learning-based architecture, which adapts to the above problems through:

- Generation of synthetic data with patterns of relationship similar to those in observed data, but not identical to the observed data, to address the issue of data scarcity^28–30^, using a Generative Adversarial Network(GAN).
- Creation of failure horizons around failure observations like in^16^, to solve the issue of data imbalance that arises while using run-to-failure datasets.
- Deployment of Long Short Term Memory (LSTM) neural networks proposed by^18^, as an alternative to statistical moments to extract temporal patterns before the application of traditional machine learning models.
- Integration of AI techniques into maintenance practices, as explored by^1^, can significantly enhance maintenance strategies. This enhancement leads to improved failure prediction accuracy, reduced costs, and increased operational efficiency.

The remaining part of the paper is organized into the methods section which is the methodology that outlines the proposed architectures. Results section, which reports the results of the implementation. Finally, the conclusion section which presents the research conclusions, which concludes the analysis, limitations, and future research directions are provided.

## Methods

### Data Collection, cleaning and prepossessing

The study utilizes Production Plant Data for Condition Monitoring retrieved from the Kaggle data repository^31^. The data is from the IMPROVE project that seeks to monitor production plant conditions for non-woven materials, predicting component conditions through 8 run-to-failure experiments^32^. Data cleaning and preprocessing included the creation of data labels and one hot encoding of the labels. Sensor readings were normalized with min–max scaling to maintain consistency in scale^33^. The final data consists of 228,416 observations in the healthy category against 8 failure observations at the end of each run. This indicates a serious data imbalance issue. Overall the data consisted of 0.01% missing cases in each column.

### Addressing data scarcity

The application of machine learning in PdM carries over the general limitations associated with ML techniques. One such limitation is the need for large data for the models to learn enough patterns to predict future values correctly^23,34,35^. With the intensified effort to minimize costs by preventing equipment breakdowns and downtime in Industry 4.0, failure instances are rare, which means longer times are needed to gather enough data to train models. This makes dealing with data scarcity one of the fundamental issues in predictive maintenance^36^. Generative Adversarial Networks (GANs) have been mentioned in other fields as a feasible solution to data scarcity^23^. This study proposes the use of GANs to generate synthetic run-to-failure data with runs similar to the collected data to make the data large enough to train ML models.

The Adversarial Networks (GANs) consist of two neural networks: the Generator (G) and the Discriminator (D) which engage in a perpetual adversarial competition, each having a specific role.

**Figure 1:**
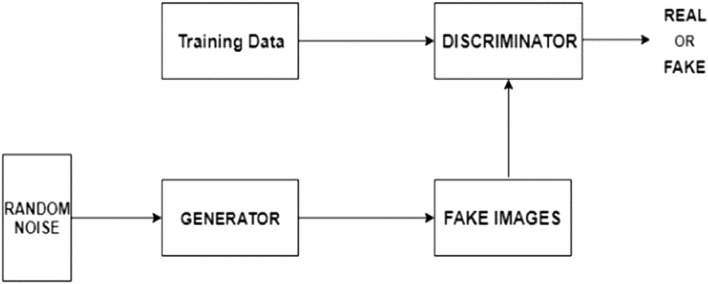
GAN Training Architecture^38^.

- Generator (G): The generator creates synthetic data. It takes a random noise vector or vector of random values as input and learns to map this input to data points that closely resemble the target dataset. Initially, the generator produces random, nonsensical data. However, it gradually refines its output through iterative training to generate data that becomes progressively more indistinguishable from the training data;
- Discriminator (D): The discriminator, on the other hand, acts as a binary classifier. It is provided with input data, which can either be real data from the training set or fake data generated by the generator. The discriminator’s task is to distinguish between these two types of data by classifying the input as real or fake. During training, the discriminator learns to become increasingly proficient at this binary classification task, thus becoming more accurate;

The hallmark of GANs is adversarial training. In this process, the generator and discriminator are trained concurrently in a mini-max game. The objective is two-fold, the generator aims to produce synthetic data virtually indistinguishable from real data. In essence, it seeks to deceive the discriminator. The discriminator, conversely, aims at correctly classifying real data as genuine and fake data as counterfeit. The adversarial competition in GAN training results in a dynamic equilibrium where the generator continually refines its ability to produce challenging data for discrimination^37^. while the discriminator refines the ability to distinguish generated data from real training data. This equilibrium signifies successful GAN training. The trained generator can therefore be used to generate synthetic data to train the ML models^38^. Figure 1 below visualizes the structure of a typical GANs model. With the GAN-generated data in hand, our methodology diverges into two primary paths: a classification path and a regression path.

### Classification path

The classification path result is the categorization of machinery state into ’healthy’ or ’failure’ classes. The healthy class denotes that the machine is in a healthy working status while failure denotes impending failure.

### Addressing data imbalance

**Figure 2:**
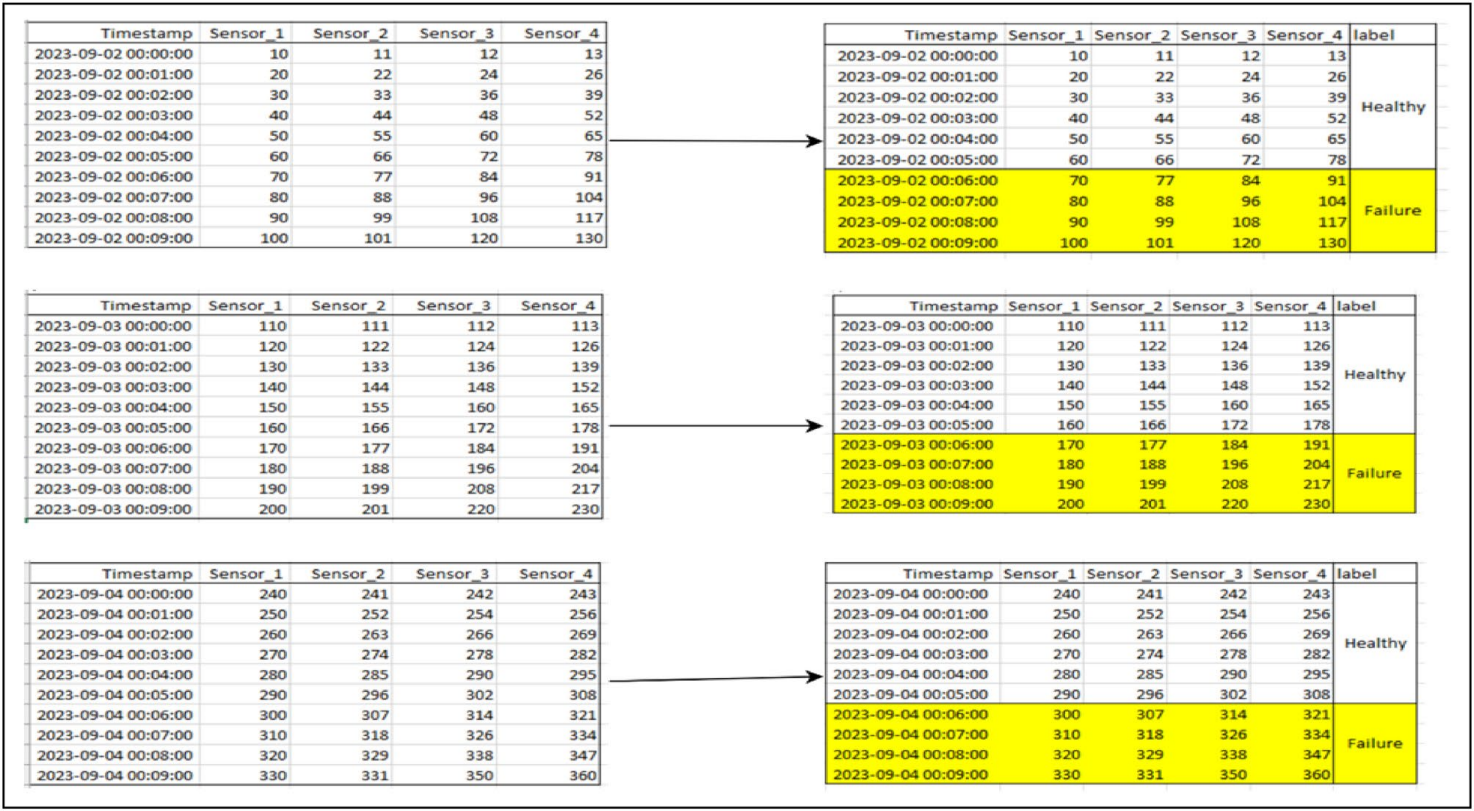
A depiction of 4- horizon categorization.

With failure conditions being rare in PdM data, owing to proactive maintenance in Industry 4.0, available datasets are highly imbalanced because the components are mostly in a healthy status rather than a failure status. In run-to-failure data, for instance, only the last observation in each run is a failure, which results in the data having many healthy cases against few failure cases. To address this issue, failure horizons were first created as proposed by Susto et al.^16^, where the component’s last ‘n’ observations before a failure event are labeled as ‘failure’, while the rest are labeled as ‘healthy’. This increases the number of failure observations in each run by ‘n’. Moreover, these horizons represent a temporal window preceding a machine failure where the machine is believed to exhibit failure patterns (signs). Figure 2 below visualizes the process of making the horizons.

This assures enough cases on the failure group for the models to learn the characteristics of a system descending to failure. The value of ‘n’ is a trainable parameter chosen via hyperparameter tuning.

### Addressing temporal dependence and feature selection

In predictive maintenance, data is collected from the same components or system continuously over time to help under- stand the component’s behavior and failure patterns, this way observations collected from the same sensor are temporally dependent^39,40^. For instance, the scenario where a bolt becomes loose within a machine:

- Vibration Sensors: Loosening bolts may cause more machine vibrations thereby resulting in severe damage. This way the readings from vibration sensors would show a multiplicative trend where the measurements of vibration increase over time. Thus, the dynamic correlation between vibration and machine health reveals that sensor readings rely on previous data.
- Temperature Sensors: Similarly, mechanical problems may influence the temperature sensors hence causing temporal dependence of previous readings and influencing the current temperature readings.

Extracting these temporal dependencies is important as it can help predict future failures. On the other hand feature selection entails scanning the entire feature space to identify an optimal feature set that doesn’t contain redundant and irrelevant features^41^. Major challenges with feature selection is the possibility of ignoring feature dependencies^42^, getting unstable features^43^ and also inability to exploit all possible feature when the feature space is large. Employing feature learning approach can help evade most of these selection problems.

Recurrent Neural Networks (RNN) are a family of Artificial Neural Networks specialized for processing sequential data such as temporal dependencies. The network features an internal state that help the model to remember past information while processing the current information^44^. A major challenge in RNNs is the vanishing gradient problem. The gradients of the loss function concerning the parameters of the network become extremely small as they are back-propagated from the output layer to the earlier layers during training^45^. Long Short-Term Memory (LSTM) networks are RNNs specialized to handle the vanishing gradient problem by having a complex memory cell instead of simple recurrent connections^46^. The memory cell features three gates which include; A forget gate that determines what information to forget from the memory cell, an input gate that determines what new information to store in the cell and an output cell that determines what information to output from the cell^46^. This gating mechanism allows gradients to flow more freely through time enabling the network to capture long-range dependencies in sequential data. The proposed methodology employed Long Short-Term Memory (LSTM) networks to extract temporal patterns from the sensor data, improving our feature set for analysis.

### Classification

The temporal features learned by the LSTM were subsequently utilized to train machine learning classifiers. A variety of classification algorithms were employed, including Artificial Neural Networks (ANN), Support Vector Machines (SVM), Decision Trees (DT), K-Nearest Neighbors (KNN), Random Forest (RF), and Extreme Gradient Boosted Classifier (XGBoost). The output of the classifiers is a classification of the component status as ’healthy’ or ’failure’, indicating whether the component is healthy or descending into failure.

### Regression path

Other than the classification path, our methodology introduces a regression-oriented approach. In this context, the focus shifts from categorizing runs into ‘healthy’ and ‘failure’ classes to predicting the Remaining Useful Life (RUL) of the machinery. RUL represents the number of useful operational days remaining before a machine is expected to fail. Because RUL is a quantitative variable, the data imbalance issue doesn’t apply.

### Addressing temporal dependence and feature selection

The regression path employs the same approach as the classification path to handle temporal dependence. LSTM architecture is utilized to learn temporal features from the GAN-generated data, which are valuable in training ML algorithms to predict the Remaining Useful Life (RUL).

### Regression models

**Figure 3:**
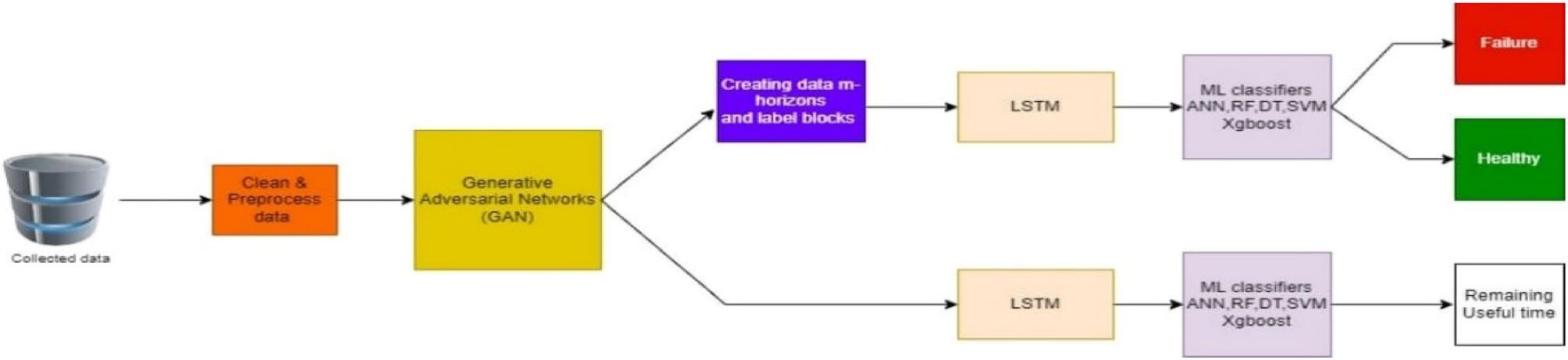
Machine learning solution architecture.

The LSTM-extracted features were passed to a suite of regression models. These models are designed to predict the Remaining Useful Life (RUL) of the machinery, providing valuable insights into how many operational days remain before potential failure. The regression models employed include: Artificial Neural Networks (ANN), Support Vector Regressor (SVR), Decision Trees Regressor (DT), K-Nearest Neighbors Regressor (KNN), Random Forest Regressor (RF), and Extreme Gradient Boosted Regressor (XGBoost). These models can quantitatively estimate the RUL of the machinery, providing maintenance personnel with actionable information on when maintenance actions should be taken. Figure 3 below visualizes the steps of the proposed methodology.

### Proposed models

#### Artificial neural networks (ANN)

Artificial Neural Networks are deep learning models capable of learning complex relationships within data. They function through three primary layers: the input layer, hidden layer, and output layer^24^. The input layer receives data and assigns activation values, while the hidden layer performs core computations and applies an activation function to standardize the output^13^. Several hidden layers can be stacked together to form a deep neural network. The output layer presents the outcome using a cost function, indicating the network’s proximity to achieving the desired result. The activation value moves from input to output nodes, where weights are adjusted and the cost function measures the difference between actual and projected output, enhancing network performance. Two key processes are introduced to minimize the cost function and enhance the network’s performance;

1. Back Propagation: This involves neural network training where weights are adjusted to boost model precision and accuracy, minimize error rates, and minimize cost function value.
2. Forward Propagation: Data enters through the input layer, and moves forward through the network to obtain the output, which is then compared to the anticipated results.

Subsequent steps encompass error computation and backward propagation, which enables weight adjustments. This structured algorithm allows for the concurrent adjustment of weights, thereby helping identify which weights contribute to errors.

### K nearest neighbour (KNN)

A KNN model is a supervised learning model that predicts new cases based on the labels of the most similar cases in the training set. The model requires users to choose a parameter K, which represents the number of neighbors to consider. It computes the distance between the new case and all cases in the training set using distance metrics such as Euclidean distance, then determines the K nearest cases to the new case. Subsequently, the new case is labeled with the most common label among the K neighbors in the case of classification^47^, or with the average of the labels of the K nearest neighbors for regression tasks^48^.

### Decision tree

In classification context DT works through binary splitting to categorize data into multiple outcomes. The process begins with a single node and proceeds to split the training data into subgroups (nodes) according to the feature most correlated with the outcome variable^49,50^. Each subgroup is then further divided based on the feature most associated with the outcome variable within those subgroups. This process continues until all nodes become pure or are unable to be split further. The primary measures of node impurity/purity are typically Gini and entropy. In regression context, splitting is similar to classification, but the splitting objective is to sum of squared residuals at the leaf node^51^.

### Random forest

Random Forest is an advancement of the decision tree, where instead of building a single decision tree that is prone to over fitting, multiple decision trees are built as ensembles from the training set, resulting in a forest of decision trees. The algorithm first takes n bootstrap samples from the training set and builds a tree on each sample^52^. To estimate model performance during training, each case is predicted by all the trees it was not involved in building, and then the most predicted class is considered as the predicted value for that case in the classification context. In the regression context, the average of the predicted values is considered as the predicted value. To predict new cases, all trees predict the new case, and then the most predicted class is considered as the prediction for classification contexts^53^, while the average of the predictions is considered as the prediction for regression cases^54^.

### Extreme gradient boosting

Like Random forests Extreme Gradient Boosting is an advancement of decision trees, which combines multiple-week learners for this case decision trees to come up with a strong predictive model, unlike decision tree where trees are build on independent bootstrap samples, in gradient boosting the trees are build sequentially^53^, where each subsequent tree is built correct errors of the previous tree the aim is to minimize loss function commonly MSE. The final prediction of the model is usually the sum of predictions from all weak learners.

### Support vector machines (SVM)

SVMs are powerful supervised learning techniques for regression, classification, and outlier detection tasks. The models present various advantages such as memory efficiency, high-dimensional effectiveness, and robustness^55^. In classification, SVMs aim to find a hyperplane or set of hyperplanes that maximize the margin between different classes of data points. These hyperplanes are used to make predictions for new data points, where the side of the hyperplane they fall on determines their class. SVMs support various kernel functions like linear, polynomial, radial basis function (RBF), and sigmoid, allowing them to handle both linearly and non-linearly separable data. The choice of the kernel function and its parameters like C (controls misclassification penalty) and gamma (affects the decision boundary’s flexibility) plays a crucial role in SVM performance and must be carefully selected^56,57^. Support Vector Regression (SVR) is a regression method that minimizes error between predicted and actual values. It can handle unbalanced datasets, multi-class classification, and probability estimation. However, it requires tuning hyperparameters, data preprocessing, and selecting appropriate kernel functions^58^. SVR is implemented in popular libraries like scikit-learn libsvm and liblinear for efficient computation.

## Results

### GAN training

**Figure 4:**
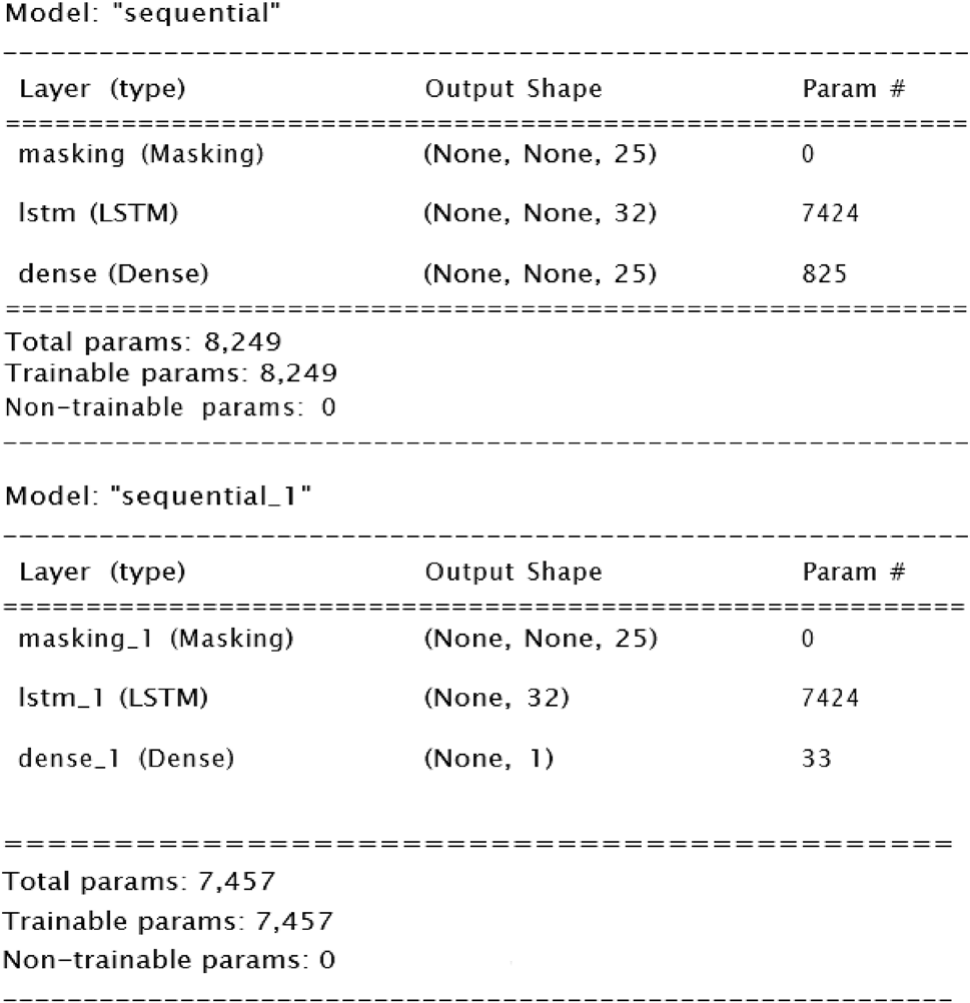
Structure of generator and discriminator models.

The GAN generator model was specified with a masking layer at the beginning to handle differing run lengths and LSTM layers to handle temporal features; the final layer includes a dense layer to control the output dimension. The discriminator included the same layers but the dense layer was binary to output classification of data as fake or real. The model was trained using a custom training loop, with batches of runs randomly sampled from the padded dataset. The discriminator classifies data as synthetic or real, while the generator aims to produce synthetic lines to deceive the discriminator. Additionally, binary cross-entropy loss functions for both the discriminator and generator were tracked throughout training. The generator minimizes this loss to improve the quality of generated sequences. Binary cross-entropy loss measures the dissimilarity between predicted probabilities and actual binary outcomes (0 or 1). It can be summarized in the formula below:

1 $$L(y,p) \, = - [y * log(p) + (1 - y) * log(1 - p)]$$

where L (y, p) is the loss for a single data point, y is the actual binary label (0 or 1), and p is the predicted probability that the data point belongs to class 1. Lower loss values for the discriminator indicate the discriminator’s ability to differentiate between synthetic and real data while for the overall model, it indicates the generator’s ability to fool the discriminator. Figure 4 below report the generator and discriminator.

**Figure 5:**
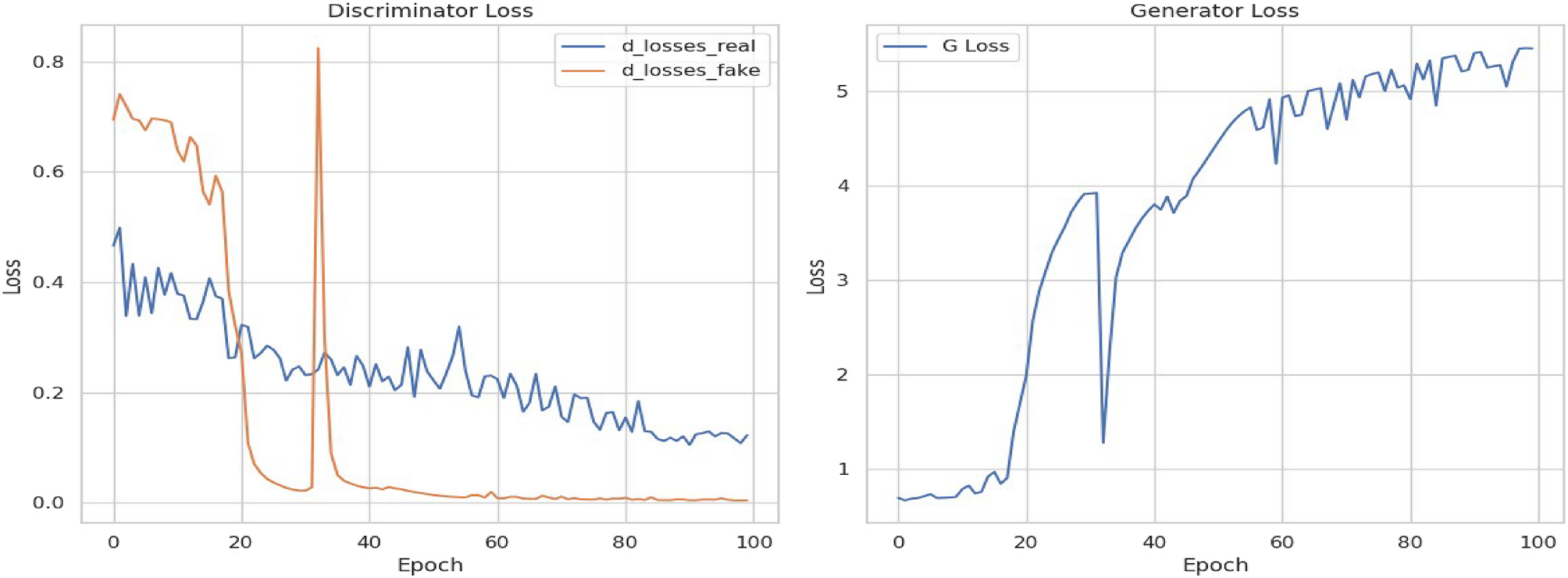
Trends in discriminator and generator loss during training.

According to the graphs in Fig. 5, the GANs framework shows a dynamic evolution of two critical loss components, that is GAN loss and discriminator loss. The discriminator initially reduces loss when acquiring real data patterns, while gradually understanding fake data characteristics. Besides, the generator loss’s trajectory varies from an upward trend in discriminator training to a descending trend, an indication that it has a growing efficiency in duping the discriminator, with a key point at equilibrium.

After training the generator, synthetic data was generated using the median run size, resulting in 20 runs. The data was normalized to match the minimum and maximum values of the collected datasets before inclusion. The study addressed the issue of data imbalance in classification models by segmenting data into healthy segments (N–n readings) and failure segments (n readings), adjusting parameter ’n’ to evaluate model performance and sensitivity to different prediction horizons. Conversely, temporal dependence was addressed by introducing LSTM neural networks where the number of LSTM layers and neurons for each layer was decided through hyperparameter tuning. The network utilized a maximum pooling and dense layer to control output feature dimensionality, while a masking layer at the top handles dataset padding.

### Classification feature extraction

**Figure 6:**
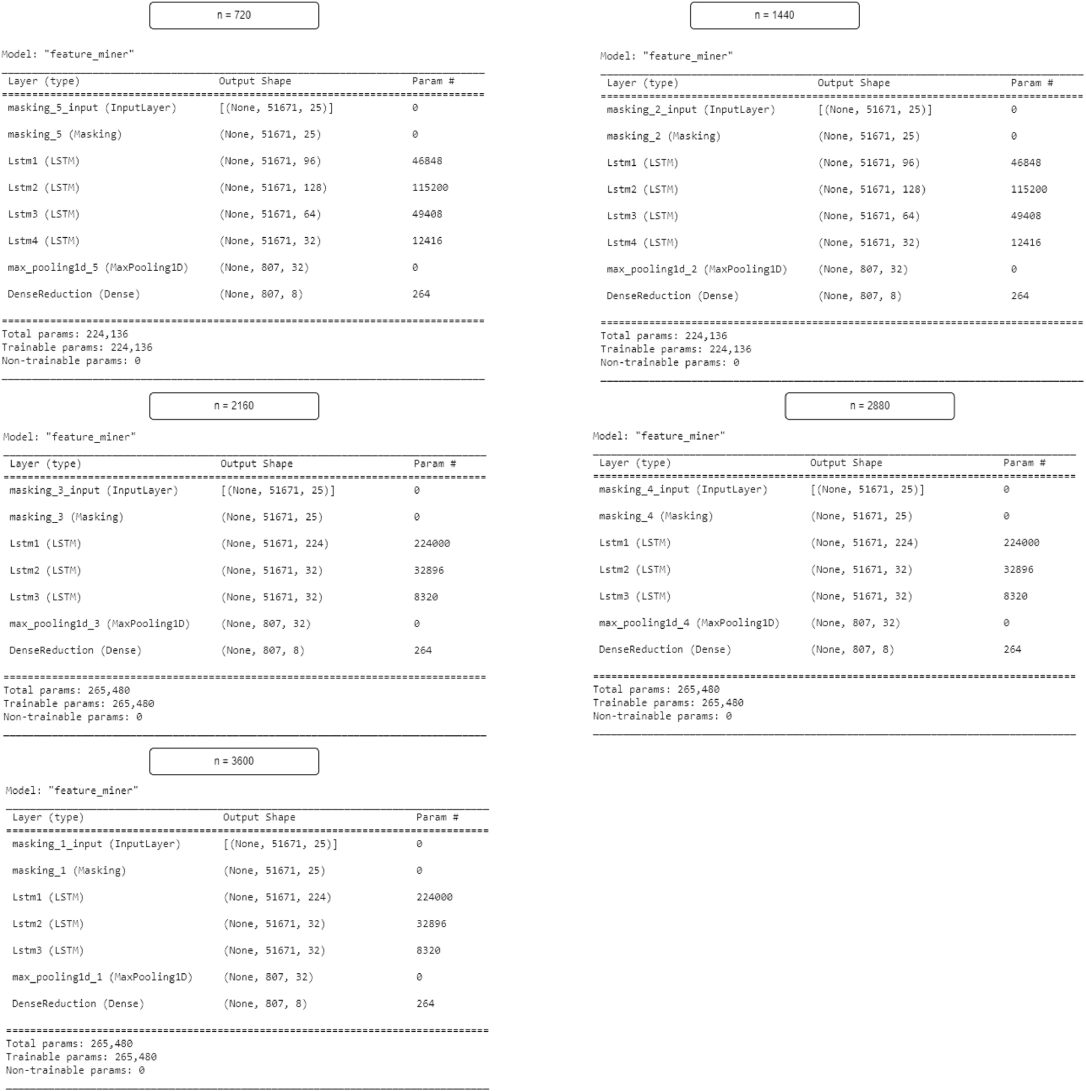
Trends in LSTM model loss.

The LSTM feature extractor for the classification path was trained jointly with the ANN classifier to learn the extraction of temporal features which can be used to classify component status. The number of LSTM layers to be used and the number of units for each layer were tuned using a Keras tuner to achieve optimal performance. Moreover, 5 horizon sizes were tried to see which horizon size was most appropriate. Figure 6 below reports optimal model for each widow size.

### ANN classifier

**Figure 7:**
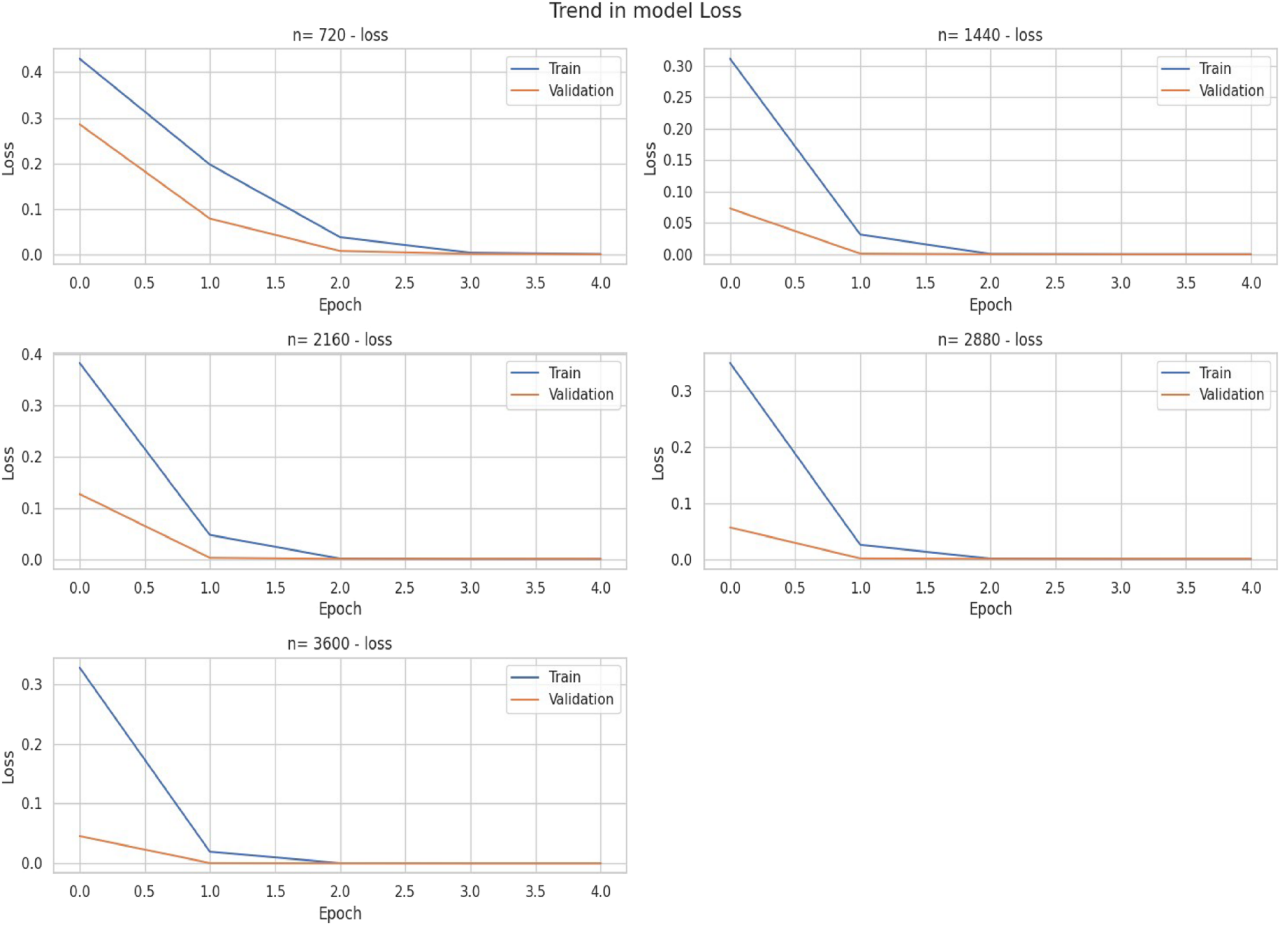
Trends in LSTM model loss.

The ANN layer uses the LSTM extracted features to classify component status with a sigmoid activation and 1 unit output. The system, combined with a binary cross-entropy loss function, quickly learns to predict component health in a few epochs but needs more training epochs for larger n-horizons. Figure 7 visualizes the trend in training and validation loss throughout the training process.

Table 1 reports the Artificial Neural Network (ANN) classification results for all the chosen *n* horizons. The model correctly predicted 88.98% of the next downtime across a horizon of 720 observations, with 99.19% of the healthy observations and 88.69% of the failure cases correctly predicted. The model precision is 99.97% which is the proportion of cases predicted as failure cases that are failures. The F1 score is 93.99% indicating that the model performs well in identifying both cases. Generally, the accuracy, sensitivity, specificity, and F1 score decrease as the horizon size increases. This indicates that the model can more accurately detect impending failures that are nearer in time compared to those farther in the future.

**Table 1:** Table of Model performance metrics.

Measure	n = 720	n = 1440	n = 2160	n = 2880	n = 3600
Accuracy	0.889826	0.878790	0.822497	0.818892	0.807892
Sensitivity	0.886903	0.871930	0.806919	0.796817	0.786915
Specificity	0.991944	0.995208	0.993565	0.995174	0.985163
Precision	0.999740	0.999676	0.999274	0.999242	0.989041
F1_score	0.939947	0.931444	0.892854	0.886623	0.989440

### Other classifiers

The extracted LSTM features were applied to baseline ML classifiers including Random Forest, XG Boost, Decision Tree, KNN, and SVM. Hyper parameters were tuned for each model using a Keras tuner. Table 2 below reports the optimal parameter setting for each classifier. Generally, model performance decreased with the horizon size, with a failure horizon of 720 observations yielding the best results for all baseline classifiers. Overall, SVM and Random Forest had the highest accuracy (74.15%) and identified failures better (specificity = 49.78%). Extreme boosted decision tree and KNN identified healthy conditions slightly better (specificity = 49.78%), while KNN had the highest precision. Support vector machine and Random Forest had the highest accuracy. Table 2 below reports this information.

**Table 2:** Optimal parameters for the classification models.

Classifier	Parameters
KNeighborsClassifier	KNeighborsClassifier(n_neighbors = 20)
DecisionTreeClassifier	DecisionTreeClassifier(max_depth = 90, max_features = ’log2’, max_leaf_nodes = 30, min_samples_leaf = 12, min_weight_fraction_leaf = 0.2)
RandomForestClassifier	RandomForestClassifier(max_depth = 50, max_features = ’auto’, max_leaf_nodes = 40, min_samples_leaf = 3, random_state = 0)
SVC	SVC(C = 5.0, gamma = ’auto’, tol = 0.1)
GradientBoostingClassifier	GradientBoostingClassifier(learning_rate = 0.02, max_depth = 15, min_samples_split = 5, n_estimators = 1000)

Generally, model performance decreased with the horizon size, with a failure horizon of 720 observations yielding the best results for all baseline classifiers. Overall, SVM and Random Forest had the highest accuracy (74.15%) and identified failures better (specificity = 49.78%). Extreme boosted decision tree and KNN identified healthy conditions slightly better (specificity = 49.78%), while KNN had the highest precision. Support vector machine and Random Forest had the highest accuracy. Table 3 below reports this information.

**Table 3:** Performance of the baseline classifiers.

Model	Performance at different horizons					
	Measure	*n* = 720	*n* = 1440	*n* = 2160	*n* = 2880	*n* = 3600
KNN	Accuracy	0.740242	0.726611	0.713910	0.700434	0.684015
	Sensitivity	0.495043	0.481722	0.469641	0.456629	0.437423
	Specificity	0.985440	0.971499	0.958178	0.944238	0.930607
	Precision	0.971429	0.944141	0.918231	0.891173	0.863081
	F1_score	0.655859	0.637949	0.621439	0.603851	0.580592
Decision tree	Accuracy	0.738228	0.727695	0.712051	0.695167	0.681382
	Sensitivity	0.491326	0.483891	0.465613	0.445787	0.429058
	Specificity	0.985130	0.971499	0.958488	0.944548	0.933705
	Precision	0.970624	0.944377	0.918143	0.889370	0.866166
	F1_score	0.652406	0.639902	0.617883	0.593892	0.573855
Random forest	Accuracy	0.741481	0.727850	0.713755	0.700434	0.686029
	Sensitivity	0.497831	0.484201	0.469641	0.456629	0.440830
	Specificity	0.985130	0.971499	0.957869	0.944238	0.931227
	Precision	0.970997	0.944411	0.917676	0.891173	0.865046
	F1_score	0.658202	0.640180	0.621311	0.603851	0.584034
Support vector machine	Accuracy	0.741481	0.727850	0.714219	0.700434	0.685564
	Sensitivity	0.497831	0.484201	0.470570	0.456629	0.441140
	Specificity	0.985130	0.971499	0.957869	0.944238	0.929988
	Precision	0.970997	0.944411	0.917825	0.891173	0.863030
	F1_score	0.658202	0.640180	0.622159	0.603851	0.583846
XG boost	Accuracy	0.739312	0.724752	0.711896	0.699814	0.680452
	Sensitivity	0.493185	0.477076	0.464684	0.454771	0.425960
	Specificity	0.985440	0.972429	0.959108	0.944857	0.934944
	Precision	0.971324	0.945365	0.919118	0.891859	0.867508
	F1_score	0.654202	0.634136	0.617284	0.602380	0.571369

### Regression feature extraction

**Figure 8:**
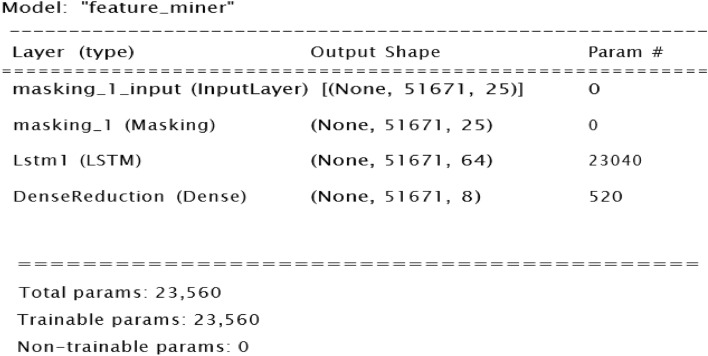
optimal feature extractor.

For the regression path, the LSTM feature extractor was trained jointly with the ANN classifier to extract temporal features appropriate for RUL prediction. The number of LSTM layers and units in each layer were tuned to get the optimal feature extractor. Figure 8 visualizes the optimal feature extractor which contains one LSTM layer of 64 units.

**Figure 9:**
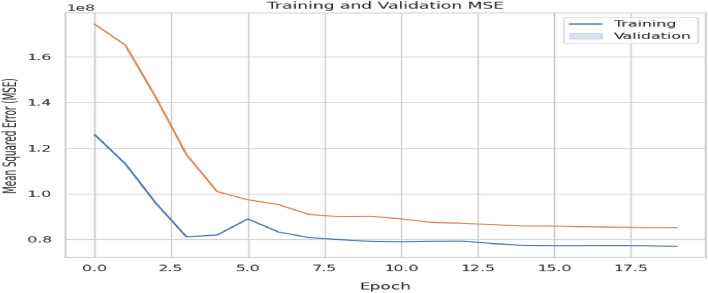
Trend in training and validation MSE.

The model was compiled with the mean squared error loss function. Figure 9 visualizes the trend in training and validation loss during training, the two drops gradually to optimal values by the 8th training epoch (Table 4).

**Table 4:** Regression models parameter settings.

Model	Parameter settings
KNeighborsRegressor DecisionTreeRegressor RandomForestRegressor SVR GradientBoostingRegressor	n_neighbors = 4751 max_depth = 70, max_features = ’sqrt’, max_leaf_nodes = 30, min_samples_leaf = 6, min_weight_fraction_leaf = 0.3 max_depth = 90, max_features = ’sqrt’, max_leaf_nodes = 10, min_samples_leaf = 12, min_weight_fraction_leaf = 0.2, random_state = 0 C = 5.0, gamma = ’auto’, kernel = ’linear’, tol = 0.1 learning_rate = 0.01, max_depth = 5, min_samples_split = 8, n_estimators = 500

### ANN regressor

A fully connected dense layer was connected to the extracted features to predict the RUL in timesteps. The model achieved MAE = 362307520.0, MSE = 1.484075e+17, RMSE = 385236896.0 and R squared − 7.657908. Table 5 compares the performance of this model with other baseline models.

**Table 5:** Regression model performance metrics.

Model	MAE	MSE	RMSE
ANN	3*.*62 × 10^8^	1*.*48 × 10^17^	3*.*85 × 10^8^
KNN	8612.42	152,786,500	12,360.69
DT	8434.81	146,647,300	12,109.80
RF	8656.18	144,307,100	12,012.79
SVR	8347.24	229,316,400	15,143.20
Xgboost	18,492.86	679,985,400	26,076.53

### Other regressors

Other regressors, including KNN, Decision Tree, Random Forest, SVR, and XGBoost regression, were fitted on the extracted features. Keras Tuner was used to optimize parameter values. Table 4 below reports the optimal parameter settings for these models. The Decision Tree model achieved the lowest RMSE among the baseline models (12109), which was even lower than the RMSE for the proposed ANN regressor.

## Conclusion and recommendations

The study has successfully implemented a machine learning-based architecture that addresses these problems by employing a generative adversarial network to resolve data scarcity, creating failure horizons to address data imbalance, and using LSTM to learn temporal patterns from the PdM dataset. In the provided case study, where data scarcity and data imbalance are evident with only 8 runs and 8 failures against 228,416 healthy observations, and where temporal dependence is present as data is collected from the same components over time, the proposed architecture adapts well to the data. It produces high accuracy even with few runs. Additionally, despite the poorly balanced data, the ANN classification results feature high F1 scores, indicating that the model can sufficiently learn patterns from both categories. Furthermore, the LSTM feature extractor successfully learns temporal patterns to classify cases and predict RUL with precision.

The interpretation of the results underscores the significance of addressing critical gaps in predictive maintenance. Firstly, the limited availability of comprehensive real-world datasets hampers the generalizability of predictive models. This highlights the urgent need for tools to compensate for data scarcity, additionally, our findings emphasize the potential of deep learning models, such as LSTM autoencoders, to capture temporal dependencies within data and enhance equipment condition predictions while avoiding manual selection of features. Lastly, the integration of AI techniques into maintenance practices is explored.

The research, holds promise in enhancing maintenance strategies, resulting in improved failure prediction accuracy, reduced operational costs, and increased operational efficiency. This highlights the significance of adopting AI technologies in Industry 4.0 settings, where optimization in maintenance practices can yield substantial benefits. In summary, our research findings indicate that addressing these gaps through collaborative data efforts, standardized metrics, advanced techniques, and AI integration can lead to more reliable, cost-effective, and efficient maintenance practices across industries.

## Limitations and future research

The research findings offer valuable insights into the critical gaps and potential advancements in predictive maintenance. However, it is essential to acknowledge certain limitations that could guide future research. Firstly, the proposed approach is computationally intensive, especially for the ANN classification where every new case has to be padded into a fixed size, before prediction, this is computationally costly for large data. Secondly, the GAN model learns to generate data similar to the provided real data. In the case of diverse Failure modes, the GANs model will not be able to generalize to patterns of failure mode it is not trained on. The study proposes Future research with more robust and diverse datasets, to test the adaptability of the architecture on diverse failure modes.

## Data Availability

The sensor data used in this study is accessible via the following link to https://www.kaggle.com/datasets/inIT-OWL/production-plant-data-for-condition-monitoring. Data analysis in this study can be provided by the corresponding author upon request.
